# Integration of Genetic and Imaging Data for Alzheimer's Disease Diagnosis and Interpretation

**DOI:** 10.1002/advs.202507629

**Published:** 2025-08-11

**Authors:** Yanfei Wang, Qing Wang, Minghao Zhou, Jialu Liang, Lei You, Breton Asken, Xiaobo Zhou, Qianqian Song

**Affiliations:** ^1^ Department of Health Outcomes and Biomedical Informatics College of Medicine University of Florida Gainesville FL 32608 USA; ^2^ Center for Computational Systems Medicine McWilliams School of Biomedical Informatics The University of Texas Health Science Center at Houston Houston TX 77030 USA; ^3^ Department of Clinical and Health Psychology University of Florida Gainesville FL 32608 USA

**Keywords:** Alzheimer's disease neuroimaging initiative, Alzheimer's disease, contrastive learning, magnetic resonance imaging, neuroimaging biomarkers, single nucleotide polymorphism, UK biobank

## Abstract

Alzheimer's disease (AD) is a progressive neurodegenerative disorder characterized by complex interactions between genetic risk factors and structural brain changes. Traditional diagnostic approaches that rely on single‐modality data, such as imaging or genomics alone, often fall short in both predictive accuracy and biological interpretability. To address these limitations, AlzCLIP, a novel contrastive learning framework that integrates single nucleotide polymorphism (SNP) profiles and MRI‐derived imaging features into a unified embedding space is introduced. This joint representation captures disease‐relevant interactions between genetic variation and brain structure, enabling both accurate diagnosis and mechanistic insight into AD. AlzCLIP is trained and evaluated on two large‐scale cohorts, the Alzheimer's Disease Neuroimaging Initiative (ADNI) and the UK Biobank (UKB), and demonstrated robust diagnostic performance, outperforming state‐of‐the‐art baselines by up to 19%. More importantly, the model yields interpretable outputs through feature importance and interaction analyses, identifying key contributors to AD risk, including rs1135173, rs7575209, and rs66763080, as well as structural markers such as hippocampal volume and precuneus surface area. Notably, AlzCLIP uncovered genotype‐specific effects on imaging phenotypes. Specifically, rs11077054 is associated with increased white matter hyperintensity burden and amygdala atrophy, suggesting a potential link between this variant and AD‐related structural brain changes. Together, the results highlight AlzCLIP's potential to enhance AD risk prediction and provide biologically grounded insights by integrating multi‐modal genomic and imaging data.

## Graphical abstract

AlzCLIP is a novel contrastive learning framework that integrates single nucleotide polymorphism (SNP) profiles and MRI‐derived imaging features into a unified representation space to improve Alzheimer's disease (AD) diagnosis and interpretation. By achieving superior diagnostic accuracy and revealing key genetic and structural contributors to AD risk, AlzCLIP provides biologically interpretable insights that illuminate the complex genotype‐phenotype interactions underlying Alzheimer's disease.

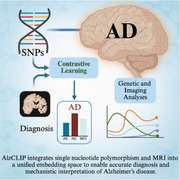

## 1 Introduction

Alzheimer's disease (AD) is a progressive neurodegenerative disorder and the leading cause of dementia,^[^ ^1^ ^]^ accounting for 60–80% of cases worldwide.^[^ ^2^ ^]^ It is characterized by a gradual decline in cognitive function, including impairments in memory, reasoning, and problem‐solving abilities. As the disease progresses, individuals experience significant neuronal loss and structural brain alterations, leading to difficulties in performing daily activities and a loss of independence. The hallmark pathological features of AD include the accumulation of amyloid‐beta (Aβ) plaques and neurofibrillary tangles, which disrupt neural communication and contribute to widespread neuronal death.^[^ ^3^ ^]^ Despite decades of research, early detection and precision diagnostics remain significant challenges, primarily due to the complex and heterogeneous nature of the disease. AD arises from a complex interplay between genetic susceptibility, environmental influences, and neurobiological changes, making accurate diagnosis particularly difficult.

Although AD is influenced by both genetic predisposition and structural brain changes, current diagnostic approaches often rely on single‐modality assessments that fail to capture the multifactorial nature of the disease. From a genetic perspective, studies such as Hong et al.’s genome‐wide association study (GWAS) on amyloid‐beta and tau‐protein species in cerebrospinal fluid (CSF) have identified novel genetic determinants (e.g., rs9877502, rs4844610, and rs744373) of AD biomarker variability.^[^ ^4^ ^]^ Similarly, Andrews et al. explored the complex genetic architecture of AD, shedding light on polygenic risk factors (e.g., rs6733839, rs9331896, and rs11218343) and their contributions to disease progression.^[^ ^5^ ^]^ From an imaging perspective, Morris et al. demonstrated the predictive power of entorhinal cortex and hippocampal measures in preclinical AD,^[^ ^6^ ^]^ while Plant et al. identified key MRI biomarkers associated with mild cognitive impairment (MCI)‐to‐AD conversion.^[^ ^7^ ^]^ While insightful, these studies often analyze genetic and imaging data separately, limiting diagnostic accuracy and personalized intervention development. Therefore, it is necessary to develop multi‐modal models that align genetic profiles with neuroimaging features, uncover their hidden associations, improve risk stratification, and enable earlier, more precise diagnoses.

In recent years, deep learning‐based AD diagnostic models have made substantial progress, particularly through the use of Convolutional Neural Networks (CNNs).^[^ ^8^, ^9^, ^10^, ^11^, ^12^, ^13^, ^14^ ^]^ CNNs have been widely employed to analyze MRI features, which are capable of extracting complex spatial patterns from imaging data, capturing subtle changes in brain structure indicative of neurodegeneration. To enhance interpretability, researchers have introduced attention‐based models, such as 3D ResNet^[^ ^15^ ^]^ and dual attention modules (3D‐DAM^[^ ^16^ ^]^). These models enable a more fine‐grained analysis of MRI scans by emphasizing key brain regions of interest (ROIs) associated with AD, such as the hippocampus, entorhinal cortex, and prefrontal cortex. While these attention‐based models improve interpretability, they fail to integrate genetic information, limiting their ability to capture the full spectrum of AD pathophysiology. Recognizing the limitations of unimodal approaches, recent studies have sought to improve the accuracy of prediction by integrating multimodal data. For instance, Gene‐SGAN^[^ ^17^ ^]^ applies a multi‐view weakly supervised clustering framework to genetic and phenotypic data to identify disease subtypes, although its design prioritizes clustering over classification. ViT‐XGBoost^[^ ^18^ ^]^ combines structural MRI and SNP data using Vision Transformers and gradient boosting for diagnostic modeling, primarily targeting mood disorders but conceptually extendable to neurodegenerative diseases. Other approaches, such as the Residual Attention Contrastive Fusion (RACF) module^[^ ^19^ ^]^ originally proposed in the context of Parkinson's disease (PD), tackle cross‐modal heterogeneity by dynamically assigning attention weights across modalities and applying contrastive loss to learn robust joint representations. These studies underscore the growing interest in multimodal data fusion; however, many existing approaches either lack a specific focus on AD or are not optimized to balance classification performance with biological interpretability.

Recent progress in contrastive learning frameworks such as Contrastive Language–Image Pre‐training (CLIP)^[^ ^20^ ^]^ has demonstrated exceptional success in aligning heterogeneous data modalities within a unified representation space. Building on these advances, we propose AlzCLIP, a novel multi‐modal framework leveraging genetic and neuroimaging data to provide a more comprehensive understanding of AD. Unlike conventional models that analyze imaging and genetic data separately, AlzCLIP employs contrastive learning to map genetic and imaging features into a unified latent space, ensuring that different data modalities are aligned in a way that reflects their disease relevance while capturing complex genotype‐phenotype interactions. By integrating genetic and neuroimaging data within a single multi‐modal framework, AlzCLIP enhances predictive accuracy and unveils hidden relationships between genetic risk factors and neuroimaging markers. This approach provides a powerful tool for early detection and personalized diagnostics, advancing precision medicine in Alzheimer's disease research.

## 2 Results

### 2.1 Overview of the AlzCLIP Model

**Figure 1:**
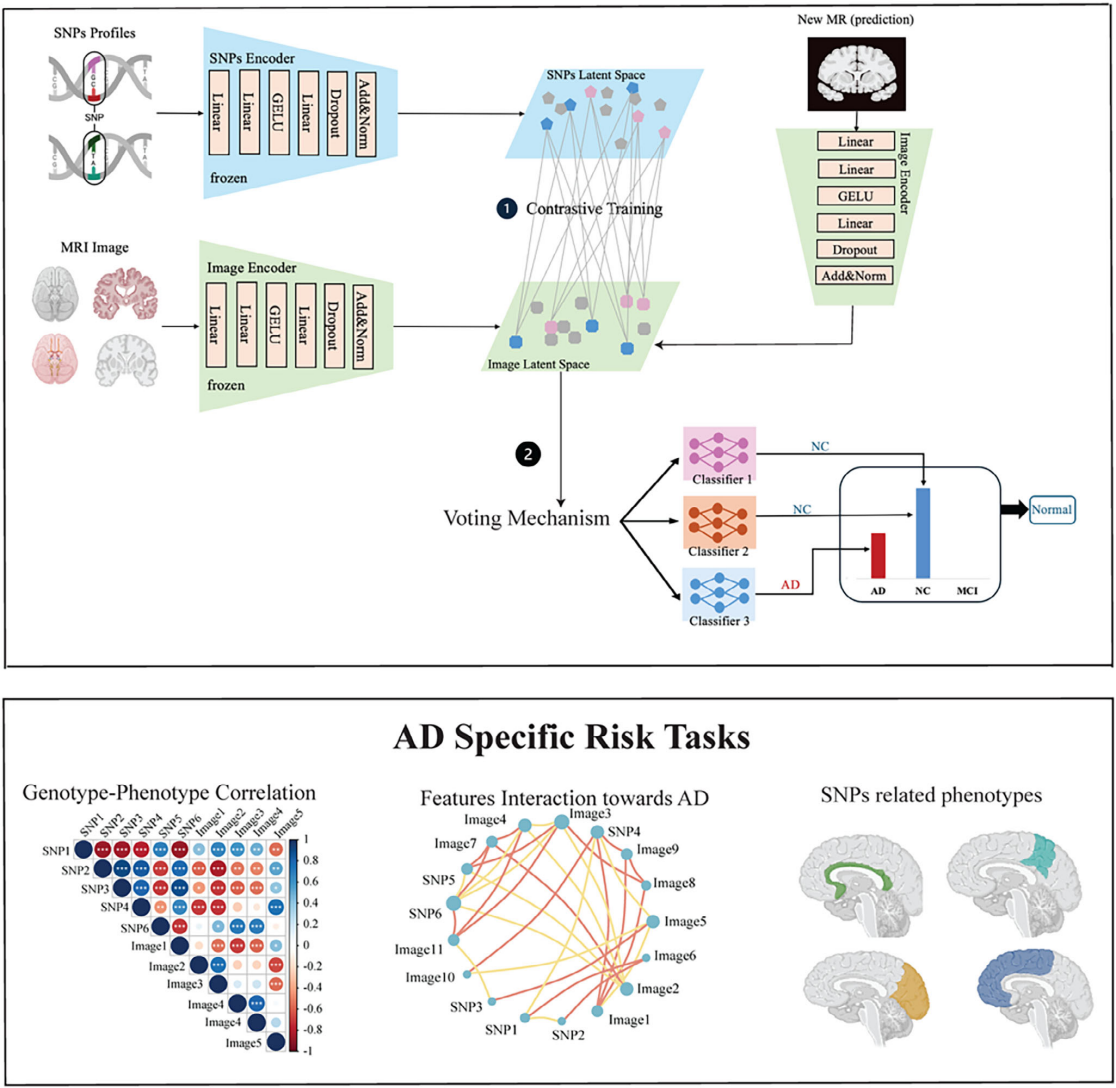
Overview of the AlzCLIP framework for AD prediction and mechanistic interpretation. A) The AlzCLIP model integrates SNP profiles and MRI images through separate encoders into a shared latent space using contrastive training. The goal is to align embeddings from the same individual while distinguishing those from different diagnostic groups (NC, MCI, AD). A downstream voting‐based ensemble classifier aggregates predictions from multiple classifiers—Random Forest, SVM, and XGBoost—using the learned embeddings, leading to robust diagnostic predictions across cognitive stages. B) AlzCLIP supports downstream analyses by uncovering genotype–phenotype associations, including the joint contributions of specific SNPs and imaging features to AD risk.

AlzCLIP is an advanced contrastive learning framework designed to integrate genetic and imaging data within a shared embedding space, facilitating the discovery of intricate cross‐modal relationships that contribute to AD progression. At the core of AlzCLIP is a contrastive training strategy that jointly optimizes representations from SNP profiles and MRI features. By aligning embeddings from the same individual and enforcing separation between those from different diagnostic groups, AlzCLIP learns a disease‐discriminative latent space that captures meaningful genotype–phenotype associations. This approach allows the model to focus on AD‐relevant genetic–structural interactions while suppressing irrelevant variability. Once trained, AlzCLIP uses the learned image and SNP embeddings for downstream classification. Rather than relying on a single prediction head, AlzCLIP employs a voting‐based ensemble mechanism, shown in **Figure** **1** as a natural extension of the contrastive embedding space. Multiple classifiers, including Random Forest, SVM, and XGBoost, operate on the unified representation to generate AD diagnostic predictions. Their outputs are aggregated through a voting scheme to yield a robust consensus diagnosis across NC, MCI, and AD categories. This ensemble strategy enhances stability and accuracy by leveraging the complementary strengths of individual classifiers. One key advantage of AlzCLIP is its ability to uncover biologically meaningful relationships between genetic variations and structural changes in the brain, enhancing the interpretability of imaging features. This not only facilitates a deeper understanding of the genetic underpinnings of AD but also helps in identifying critical imaging and genetic biomarkers associated with disease progression. Therefore, AlzCLIP serves as a powerful tool for investigating multi‐modal interactions in neurodegenerative diseases, offering both enhanced diagnostic accuracy and deeper biological insights into AD.

### 2.2 AlzCLIP Demonstrates Superior Performance in AD Diagnosis

To evaluate the predictive performance of AlzCLIP in AD diagnosis, we compared it with established baseline methods under three experimental settings: SNP‐only, MRI‐only, and combined MRI and SNP data. In the MRI‐only setting, AlzCLIP was benchmarked against 3D‐DAM,^[^ ^16^ ^]^ 3D‐ResNet,^[^ ^15^ ^]^ and a standard convolutional neural network (CNN).^[^ ^8^ ^]^ For the SNP‐only setting, comparisons were made with CNN^[^ ^21^ ^]^ and traditional machine learning algorithms, including Support Vector Machine (SVM), Random Forest (RF), and XGBoost, all of which also constitute core components of AlzCLIP's ensemble voting strategy. In the multi‐modal setting, we included Gene‐SGAN,^[^ ^17^ ^]^ ViT‐XGBoost,^[^ ^18^ ^]^ and Contrastive Fusion (RACF) module.^[^ ^19^ ^]^ All experimental settings were evaluated on two large‐scale cohorts, ADNI and UKB.

**Figure 2:**
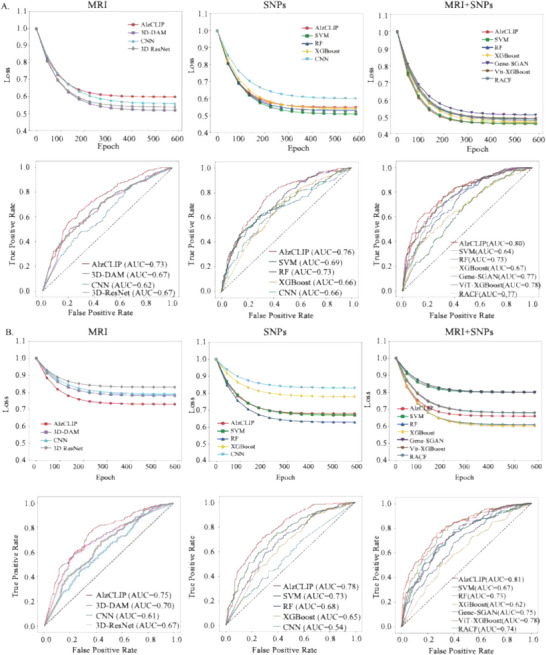
Performance Evaluation of the AlzCLIP Model. A) Model performance on the ADNI dataset (n=758; 214 AD, 180 MCI, 364 CN) across three experimental configurations: MRI‐only (left), SNP‐only (center), and combined MRI+SNP (right). Upper panels display training loss curves over 600 epochs, demonstrating convergence dynamics. Lower panels present receiver operating characteristic (ROC) curves with area under the curve (AUC) values for each model configuration. AlzCLIP (red line) achieved AUC values of 0.73 (MRI‐only), 0.76 (SNP‐only), and 0.80 (combined MRI+SNP). B) Model performance on the independent UKB validation cohort (n=261; 61 AD, 200 CN) with identical experimental design. AlzCLIP demonstrated AUC values of 0.75 (MRI‐only), 0.78 (SNP‐only), and 0.81 (combined MRI+SNP). Comparative baselines included imaging‐based methods (3D‐DAM, 3D‐ResNet, CNN), genetic‐based methods (SVM, Random Forest, XGBoost), and multimodal approaches (Gene‐SGAN, ViT‐XGBoost, RACF). The combined MRI+SNP configuration yielded optimal discriminative performance across both cohorts.

As shown in **Figures** **2**A and S1 (Supporting Information), AlzCLIP achieved superior performance across all experimental settings in the ADNI cohort. In the MRI‐only configuration, AlzCLIP obtained a final loss of 0.60, an accuracy of 0.62, and an AUC of 0.73, outperforming comparative imaging‐based models, including 3D‐DAM (loss: 0.52, accuracy: 0.48, AUC: 0.67), 3D‐ResNet (loss: 0.54, accuracy: 0.47, AUC: 0.67) and CNN (loss: 0.56, accuracy: 0.46, AUC: 0.62). In the SNP‐only setting, AlzCLIP achieved a final loss of 0.55, an accuracy of 0.6, with an AUC of 0.76, surpassing other models such as CNN (loss: 0.60, accuracy: 0.46, AUC: 0.66), SVM (loss: 0.51, accuracy: 0.58, AUC: 0.69), RF (loss: 0.53, accuracy: 0.57, AUC: 0.73), and XGBoost (loss: 0.54, accuracy: 0.56, AUC: 0.66). The highest predictive capability was observed under the combined MRI+SNP scenario, where AlzCLIP achieved a final loss of 0.46, an accuracy of 0.81 and an AUC of 0.80, substantially exceeding the multimodal baseline Gene‐SGAN (loss: 0.46, accuracy: 0.78, AUC: 0.77), ViT‐XGBoost (loss: 0.49, accuracy: 0.70, AUC: 0.78) and RACF (loss: 0.47, accuracy: 0.65, AUC: 0.77), as well as SVM (loss: 0.51, accuracy: 0.65, AUC: 0.64), Random Forest (loss: 0.49, accuracy: 0.64, AUC: 0.73), and XGBoost (loss: 0.48, accuracy: 0.63, AUC: 0.67).

This performance superiority was consistently confirmed in the UKB cohort, as demonstrated in Figure 2B and Figure S2 (Supporting Information). Under the MRI‐only condition, AlzCLIP achieved a final loss of 0.73, an accuracy of 0.73 and an AUC of 0.75, clearly surpassing imaging‐based methods such as 3D‐DAM (loss: 0.78, accuracy: 0.67, AUC: 0.70), 3D‐ResNet (loss: 0.83, accuracy: 0.67, AUC: 0.67) and CNN (loss: 0.79, accuracy: 0.70, AUC: 0.61). Similarly, in the SNP‐only scenario, AlzCLIP maintained robust performance with a final loss of 0.68, an accuracy of 0.65, and an AUC of 0.78, outperforming conventional SNP‐based approaches including CNN (loss: 0.83, accuracy: 0.62, AUC: 0.54), SVM (loss: 0.67, accuracy: 0.63, AUC: 0.73), RF (loss: 0.63, accuracy: 0.52, AUC: 0.68), and XGBoost (loss: 0.78, accuracy: 0.57, AUC: 0.65). The combined MRI+SNP configuration again delivered the strongest predictive outcomes, where AlzCLIP reached a final loss of 0.66, an accuracy of 0.79 and an AUC of 0.81, outperforming the multimodal baseline Gene‐SGAN (loss:0.80, accuracy: 0.73, AUC: 0.75), ViT‐XGBoost (loss: 0.61, accuracy: 0.78, AUC: 0.78) and RACF (loss: 0.6, accuracy: 0.75, AUC: 0.74) as well as SVM (loss:0.80, accuracy: 0.67, AUC: 0.67), Random Forest (loss:0.68, accuracy: 0.64, AUC: 0.73), and XGBoost (loss:0.68, accuracy: 0.68, AUC: 0.62).

### 2.3 AlzCLIP Enhances Multi‐Modal Feature Interpretability

**Figure 3:**
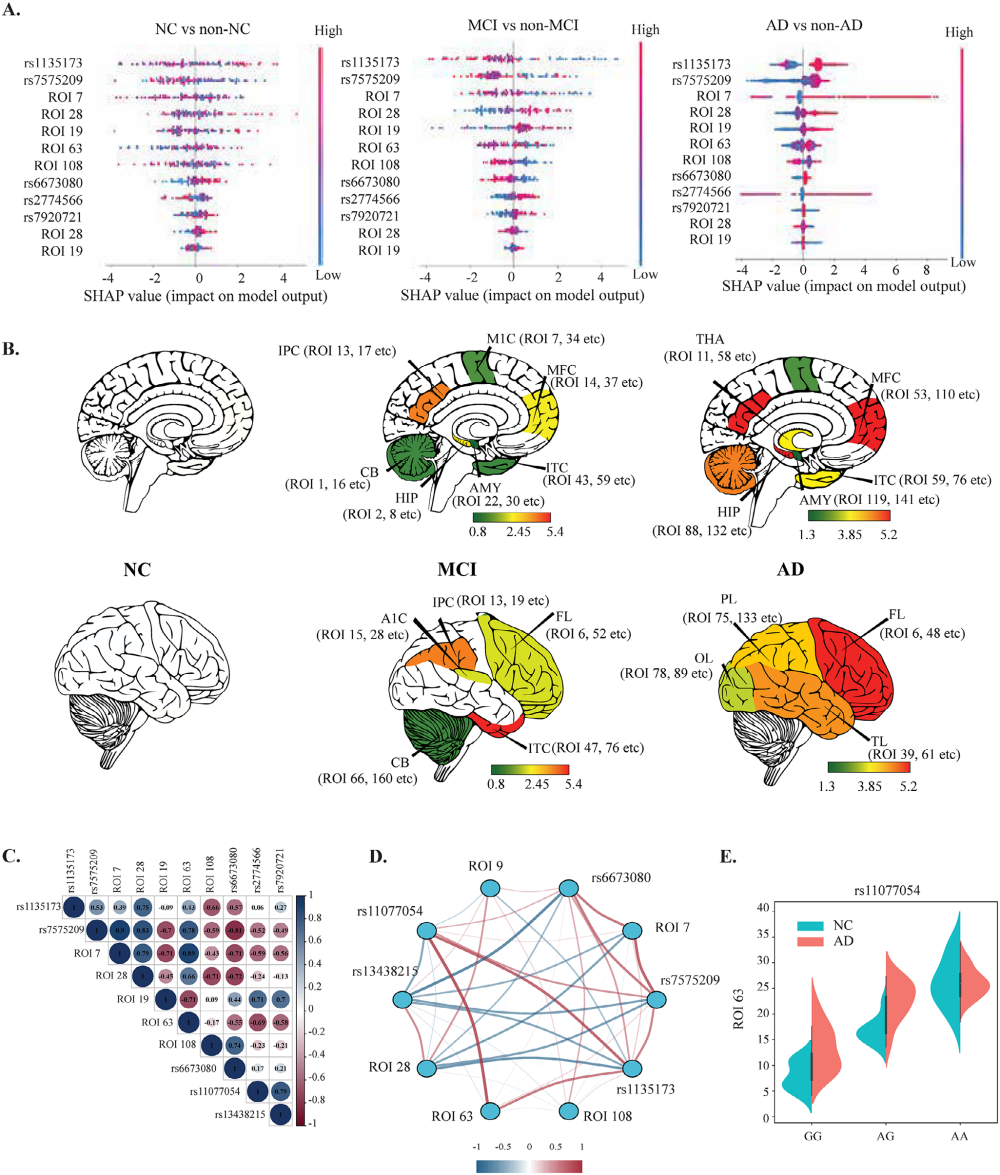
Interpretability of AlzCLIP predictions in the ADNI dataset (*n* = 758). A) SHAP summary plots showing feature importance across three diagnostic comparisons: NC vs non‐NC (left), MCI vs non‐MCI (center), and AD vs non‐AD (right). SHAP values quantify the impact of genetic variants (rs1135173, rs7575209, rs66763080, rs11077054) and neuroimaging features (ROI 19, ROI 28, ROI 63, ROI 108, ROI 76) on model predictions. Data points are colored by feature values (blue=low, red=high) and positioned by SHAP impact magnitude on the x‐axis. B) Brain maps illustrating the spatial distribution of important neuroimaging features identified by SHAP analysis across cognitive stages. The upper row shows sagittal views, lower row shows lateral views for the NC, MCI, and AD groups. Color intensity reflects SHAP magnitude with numerical scale bars provided. Anatomical regions labeled include Inferior Parietal Cortex (IPC), Anterior Insular Cortex (AIC), Inferior Temporal Cortex (ITC), Frontal Lobe (FL), Amygdala (AMY), Hippocampus (HIP), and Cerebellum (CB). C) Pearson correlation matrix displaying pairwise associations between genetic variants and neuroimaging features. Color scale represents correlation coefficients (blue=negative, white=neutral, red=positive correlation). D) SHAP interaction network showing joint feature contributions. Nodes represent individual features, edges represent interactions, with colors and line thickness indicating interaction strength according to the provided scale. E) Violin plots showing ROI 63 (Frontal Lobe) volume distributions stratified by rs11077054 genotype (GG, AG, AA) and diagnostic groups (NC, AD). Distribution density curves show data spread for each genotype‐diagnosis combination (*p* < 0.05).

To elucidate key contributors to AD prediction, we applied SHapley Additive exPlanations (SHAP^[^ ^22^ ^]^) to interpret AlzCLIP's outputs on the ADNI dataset. As shown in **Figure** **3**A, SHAP values were analyzed across three cognitive stages, i.e., NC, MCI, and AD. In the NC group, early indicators of cognitive vulnerability included genetic variants rs1135173 and rs7575209, as well as neuroimaging markers such as ROI 19 (Inferior Parietal Cortex, IPC), ROI 28 (Anterior Insular Cortex, AIC), and ROI 63 (Frontal Lobe, FL). These features exhibited moderate SHAP values, suggesting their relevance in identifying individuals at risk for progression. As the disease advanced to the MCI stage, these features retained their impact, and rs66763080 emerged as an additional contributor. Neuroimaging involvement expanded to include ROI 108 (IPC) and ROI 76 (Inferior Temporal Cortex, ITC), reflecting broader cortical disruption. Structural changes also appeared in the Cerebellum (CB) and ITC, indicating early neurodegenerative effects. These patterns are visualized in Figure 3B, where the spatial distribution of SHAP values illustrates progressive structural involvement from NC to AD. By the AD stage, both genetic and imaging markers showed increased SHAP importance. In addition to previously highlighted SNPs, rs11077054 emerged prominently, and structural degeneration extended to the Parietal (PL), Temporal (TL), and Occipital Lobes (OL). Our analysis revealed particularly strong contributions from ROI 63 (FL) and rs11077054, suggesting that individuals with high‐risk genotypes may be particularly vulnerable to frontal lobe atrophy, which is a hallmark of cognitive decline in AD.^[^ ^23^ ^]^

To further explore these relationships, we examined the correlation of the contributive features (Figure 3C), capturing pairwise statistical associations between key SNPs and neuroimaging features. Strong correlations were observed between rs1135173, rs7575209, and structural markers including ROI 28 (AIC), ROI 63 (FL), and ROI 108 (IPC), suggesting potential shared pathways underlying both genetic risk and anatomical change. Figure 3D presents SHAP interaction values, which quantify how feature pairs jointly contribute to the model's AD prediction beyond their individual effects. These values reflect the degree to which combinations of genetic variants and neuroimaging markers interact within the model to influence classification outcomes. Among the strongest interactions observed were those between rs11077054 and ROI 63 (FL) and between rs7575209 and ROI 28 (AIC). These model‐derived interactions suggest that the predictive effect of certain brain regions may be enhanced or attenuated depending on the presence of specific genetic variants. Notably, rs11077054, located in the ANKRD55 gene, which is an immune‐related gene implicated in neuroinflammatory processes,^[^ ^24^ ^]^ showed a strong model‐derived interaction with frontal lobe atrophy. Next, the violin plot in Figure 3E stratifies ROI 63 volumes by rs11077054 genotypes. Individuals with the AA genotype exhibited significantly lower ROI 63 (FL) volume in the AD group compared to AG and GG carriers. This genotype‐dependent structural vulnerability supports the hypothesis that rs11077054 plays a mechanistic role in region‐specific neurodegeneration.

### 2.4 AlzCLIP Reveals Common SNP‐Related Neuroimaging Features Across Different Cohorts

**Figure 4:**
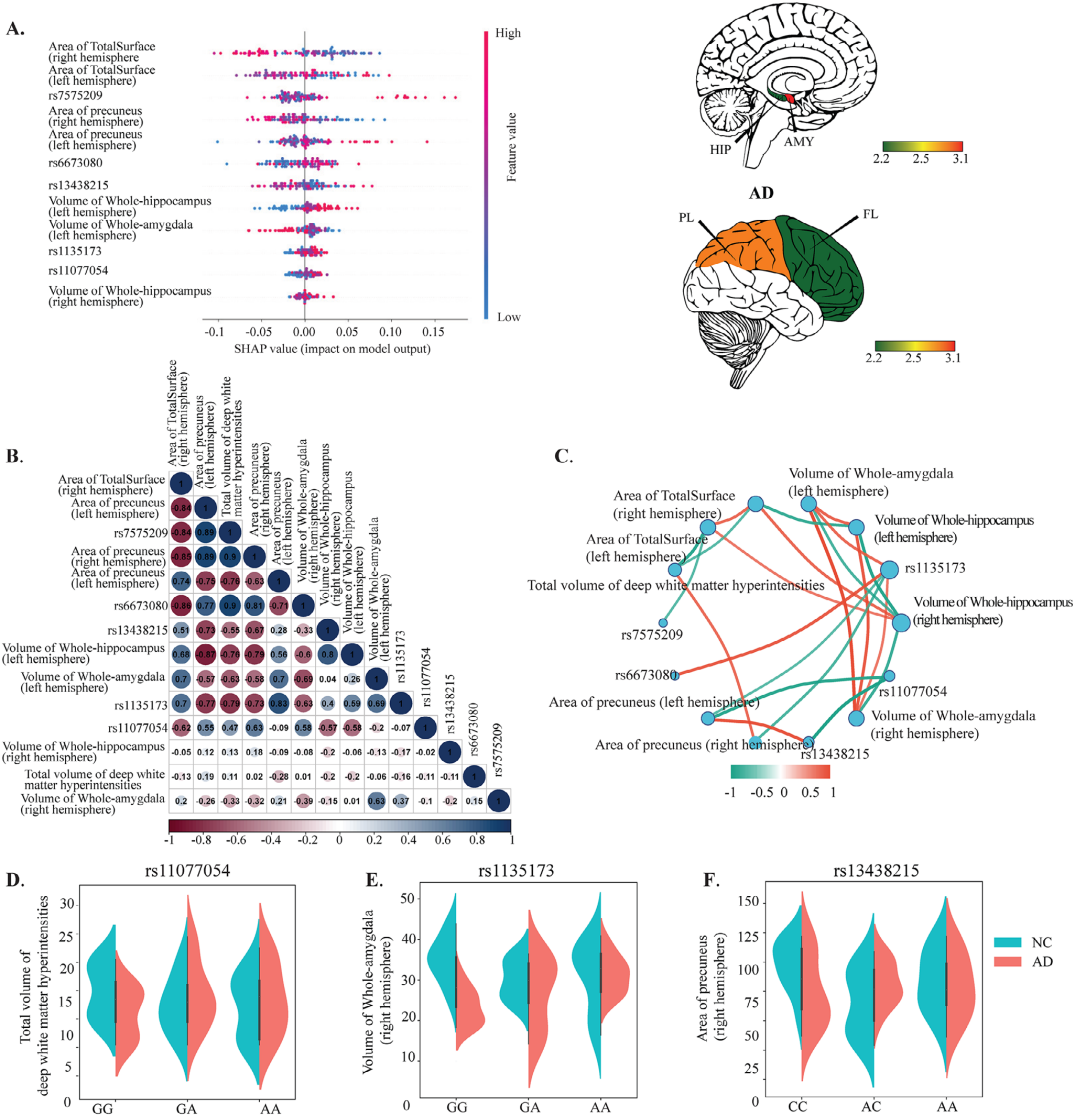
Interpretability of AlzCLIP predictions in the UKB dataset (n=261). A) SHAP summary plot displaying feature importance for AD vs NC classification. Features include genetic variants (rs11077054, rs7575209, rs66763080, rs1135173, rs13438215) and neuroimaging measures (hippocampal volume, total cortical surface area, precuneus area, amygdala volume, white matter hyperintensity volumes). Data points are colored by feature values (blue=low, red=high) and positioned by SHAP impact magnitude on the x‐axis. Brain map (right) shows spatial distribution with color scale indicating SHAP values. B) Pearson correlation matrix showing pairwise associations between genetic variants and neuroimaging features. Circle size indicates correlation magnitude, with color scale representing correlation coefficients (blue=negative, white=neutral, red=positive). Feature labels include all genetic variants and imaging measures analyzed. C) SHAP interaction network displaying joint contributions between features. Nodes represent individual features (genetic variants in pink, imaging features in blue), with edges showing interactions. Edge colors and thickness indicate interaction strength according to the provided scale (−0.5 to 0.5). D) Violin plot showing deep white matter hyperintensity volume distributions stratified by rs11077054 genotype (GG, AG, AA) across diagnostic groups (NC=cyan, AD=red) (*p* < 0.05). E) Violin plot showing precuneus area distributions by rs13438215 genotype (G, GG, AA) across diagnostic groups (*p* < 0.05). (F) Violin plot showing amygdala volume distributions by rs1135173 genotype (CC, AC, AA) across diagnostic groups (*p* < 0.05).

To evaluate the reproducibility of AlzCLIP's interpretability across cohorts, we applied the model to the UKB dataset and assessed whether the key features identified in ADNI were also prioritized in this UKB cohort. As shown in **Figure** **4**A, the top contributors to AD prediction included both genetic variants (e.g., rs11077054, rs7575209, and rs66763080) and neuroimaging markers, such as hippocampal volume, total cortical surface area, and precuneus area. These features yielded high SHAP values, underscoring their importance in distinguishing individuals with AD from healthy controls. Notably, hippocampal atrophy and cortical surface reductions are well‐established neurodegenerative markers in AD, while the precuneus, a critical hub within the default mode network,^[^ ^25^ ^]^ was again prominently implicated, which is consistent with its early involvement in cognitive dysfunction. To contextualize these neuroimaging markers, we mapped them to their anatomical regions. To contextualize these neuroimaging markers, we mapped them to their corresponding anatomical regions. Imaging features with high importance were localized to the frontal lobe, parietal lobe, hippocampus, and amygdala. These regions were also prominently identified in the ADNI analysis, reinforcing the biological plausibility and consistency of AlzCLIP's imaging‐based predictions across datasets.

We next assessed the relationships among the top features using a correlation heatmap (Figure 4B). rs11077054 showed strong correlations with hippocampal volume, amygdala volume, and white matter hyperintensity (WMH) burden, mirroring its pattern in ADNI and highlighting its broad involvement in neurodegenerative processes. Similarly, rs7575209 and rs66763080 exhibited notable correlations with cortical surface area and subcortical atrophy, consistent with their associations observed in ADNI and suggesting shared genetic modulation of brain structure across cohorts. We further examined the synergistic effects between genetic variants and imaging features (Figure 4C). The strongest interactions were observed between rs11077054 and both amygdala volume and WMH burden, indicating that the predictive influence of these neuroimaging markers may be modulated by the presence of this immune‐related variant. Additional interactions, such as rs7575209 with the precuneus area and rs66763080 with hippocampal volume, highlight specific SNP–ROI combinations that contribute disproportionately to AD diagnosis. These interaction patterns are consistent with those observed in the ADNI cohort, suggesting that AlzCLIP captures robust genotype–phenotype relationships that generalize across diverse populations.

The genotype‐specific impact of key variants was further examined in the UKB cohort. For rs11077054, individuals carrying the AA genotype exhibited significantly higher white matter hyperintensity (WMH) volumes, particularly among AD cases (Figure 4D). This finding reinforces our observations from the ADNI cohort and suggests that rs11077054 may contribute to cerebrovascular pathology, a known driver of cognitive impairment in AD. As shown in Figure 4E, the precuneus surface area demonstrated genotype‐dependent variation associated with rs13438215, with AD patients carrying the AA genotype exhibiting significantly greater cortical thinning. Similarly, Figure 4F shows that amygdala volume varied significantly across rs1135173 genotypes. Although this SNP was previously identified in the ADNI analysis, the UKB‐based violin plot provides independent validation, revealing a consistent pattern of greater amygdala atrophy in individuals with the AA genotype. This further supports its role in AD‐associated neurodegeneration.

### 2.5 Mechanistic Insights into Alzheimer's Disease Progression

**Figure 5:**
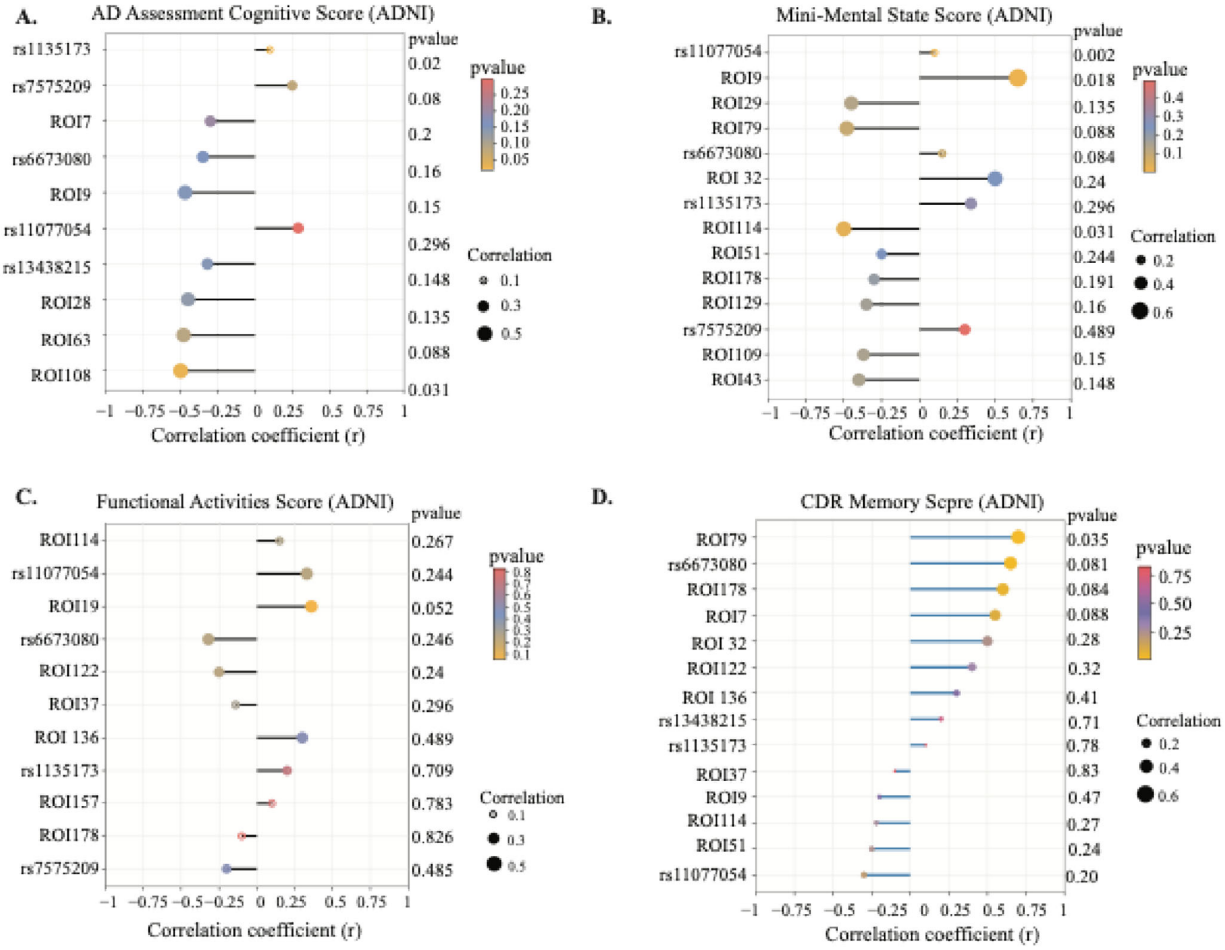
Associations between AlzCLIP‐identified features and cognitive, functional, and structural phenotypes in the ADNI dataset (n=758). A) Dot plot showing Pearson correlation coefficients between selected genetic variants (rs1135173, rs7575209, rs11077054, rs1343821, rs6129, rs1135, rs66763080) and neuroimaging features (ROI7, ROI108, ROI9, ROI108, ROI7) with AD Assessment Cognitive Score. Point size indicates correlation strength (r), color represents p‐value significance (yellow=low p‐value, blue=high p‐value) with scale bar provided. Correlation coefficients range from ≈−0.5 to +0.5. B) Dot plot showing associations between the same features and Mini‐Mental State Examination (MMSE) scores. Feature ranking and statistical encoding follow the identical format to panel A. C) Dot plot displaying correlation patterns between selected features and Functional Activities Questionnaire (FAQ) scores, where correlations reflect functional impairment associations. D) Dot plot illustrating correlations between genetic and neuroimaging features and Clinical Dementia Rating (CDR) memory scores. For all panels, statistical significance is assessed using Pearson correlation analysis with two‐tailed tests. Point size reflects correlation magnitude (larger points indicate stronger correlations), and color intensity represents statistical significance according to the provided p‐value scale (0.001–1.0). Individual correlation coefficients (r) and p‐values are listed adjacent to each feature.

To further investigate the biological significance of features identified by AlzCLIP, we examined their associations with cognitive and clinical phenotypes across the ADNI and UKB datasets. These analyses provide insight into how genetic variants and neuroimaging markers contribute to Alzheimer's disease (AD) progression. In the ADNI dataset (**Figure** **5**A–D), we assessed correlations between key SNPs and cognitive outcomes. Several variants, including rs1135173, rs66763080, and rs11077054, demonstrated significant associations with AD Assessment Cognitive Score (Figure 5A), Mini‐Mental State Examination (MMSE) (Figure 5B), Functional Activities Questionnaire (FAQ) Score (Figure 5C), and Clinical Dementia Rating (CDR) memory score (Figure 5D). Several genetic variants exhibited statistically significant associations with cognitive performance. For instance, rs1135173 was significantly correlated with the AD Assessment Cognitive Score (*p* = 0.02). Similarly, rs11077054 showed consistent associations across multiple domains, including the CDR memory score and MMSE (*p* = 0.002). Notably, several neuroimaging ROIs also demonstrated significant or trending associations with cognitive scores. ROI19 and ROI7, for instance, were consistently implicated across multiple clinical measures, reinforcing their potential as intermediate imaging biomarkers reflecting disease burden. The correlation patterns across modalities and scores support the relevance of AlzCLIP's selected features and suggest that the model is capable of identifying biologically interpretable factors linked to cognitive decline in AD.

## 3 Discussion

AD is a multifaceted neurodegenerative disorder driven by intricate interactions among genetic, molecular, and environmental factors. A comprehensive understanding of these interactions is essential for improving disease prediction and identifying therapeutic targets. In this study, we introduced AlzCLIP, a contrastive learning‐based, multi‐modal framework inspired by the CLIP model, designed to integrate genetic and neuroimaging data into a unified, interpretable representation for AD prediction and analysis. Comprehensive evaluations on two large‐scale datasets, ADNI and UKB, demonstrated the robust performance of AlzCLIP. While the model performed well using SNP‐only or MRI‐only inputs, its highest AUC was achieved in the combined SNP+MRI configuration, consistently outperforming existing state‐of‐the‐art approaches. This demonstrates AlzCLIP's effectiveness in leveraging complementary genetic and neuroimaging information to enhance AD classification.

Beyond its performance, AlzCLIP also facilitates interpretability by uncovering biologically meaningful relationships between genetic variants and brain structural changes. This integrative approach provides mechanistic insights into genotype–phenotype interactions and opens new avenues for understanding disease pathways. A notable example is the consistent association of rs11077054 with imaging markers across both the UKB and ADNI cohorts. Located in the ANKRD55 gene,^[^ ^26^ ^]^ which encodes an ankyrin repeat domain‐containing protein involved in immune regulation,^[^ ^24^ ^]^ rs11077054 has previously been implicated in multiple immune‐mediated conditions—pathways that are increasingly recognized as contributors to AD pathogenesis.^[^ ^27^, ^28^ ^]^ Our analyses revealed that rs11077054 is significantly associated with ROI 63 (frontal lobe) and total deep white matter hyperintensity (WMH) volume, both of which are key neuroimaging markers linked to executive dysfunction and cerebrovascular pathology in AD. Given the established role of WMHs as indicators of small vessel disease and neuroinflammation,^[^ ^29^ ^]^ these findings suggest that rs11077054‐mediated immune dysregulation may contribute to vascular dysfunction, thereby accelerating cognitive decline and disease progression. This highlights the potential of AlzCLIP not only as a diagnostic tool but also as a platform for generating testable hypotheses about molecular mechanisms underlying AD.

Beyond rs11077054, rs7575209 also emerged as a shared genetic risk variant across both the UKB and ADNI cohorts, demonstrating a strong and consistent association with ROI 28, located in the anterior insular cortex (AIC). The AIC is a critical node within the salience network, which plays a central role in attentional control, cognitive flexibility, and emotional regulation.^[^ ^30^ ^]^ Atrophy in this region has been linked to cognitive impairment and neuropsychiatric symptoms in AD.^[^ ^31^ ^]^ The observed association between rs7575209 and structural decline in the AIC suggests a potential genetic basis for salience network dysfunction, which could contribute to early attentional deficits and affective disturbances in AD.^[^ ^32^ ^]^ In addition, SHAP interaction analysis revealed that rs1135173 exhibited strong interactions with ROI 19, corresponding to the inferior parietal cortex (IPC), in contributing to AD prediction. The IPC is known for its role in episodic memory retrieval and visuospatial processing,^[^ ^33^ ^]^ and atrophy in this region represents one of the earliest cortical changes observed in AD.^[^ ^34^, ^35^, ^36^, ^37^, ^38^ ^]^ The high interaction value between rs1135173 and IPC suggests that their combined presence may amplify the model's prediction of AD, supporting the notion that genetically influenced alterations in parietal connectivity are central to disease mechanisms. Similarly, rs66763080 demonstrated a strong interaction with ROI 108 (also located in the IPC), further implicating this variant in parietal lobe‐related vulnerability.

These findings underscore the value of integrating SNPs and neuroimaging markers to gain a more comprehensive understanding of AD progression. Unlike traditional approaches that analyze genetic and imaging modalities in isolation, AlzCLIP employs contrastive learning to align these heterogeneous data types within a shared representation space, revealing biologically meaningful patterns that may remain hidden in single‐modality analyses. However, the limited availability of comprehensive datasets containing both high‐quality genetic and neuroimaging data remains a key challenge. Many cohorts include only one modality or have relatively small sample sizes with paired data, thereby limiting the statistical power and generalizability of AlzCLIP. In addition, though AlzCLIP has demonstrated promising results by integrating SNP and neuroimaging features, it currently treats each feature independently. Future work using network‐based models with interactions between SNPs and imaging features as input would further enhance the capture of the complex interactions between them. Thereby revealing the coordinated networks of interacting genetic and neuroimaging factors.

## 4 Experimental Section

As illustrated in Figure 1, AlzCLIP integrated imaging and genetic data into a shared embedding space for identifying AD, Cognitively Normal (NC), and Mild Cognitive Impairment (MCI) conditions. The approach consisted of a training stage where the embedding space was learned via contrastive learning, and an inference stage where the embeddings were used for diagnosis. Because SNP genotypes were time‐invariant, each individual's SNP profile and diagnostic label were linked to their MRI‐derived imaging features. This created a dataset of 2*N* MRI‐SNP and MRI‐label pairs, where *N* is the number of individuals. The dataset was represented as two feature sets: the image feature set. ${\left\{I_{t};\;I_{t}\varepsilon R^{d_{i}}\right\}}_{t=1}^{N}$ and the SNP‐label feature set ${\left\{S_{t};\;S_{t}\varepsilon R^{d_{s}}\right\}}_{t=1}^{N}$. The data was split into training (80%), validation (10%), and testing (10%) sets. Two parallel embedding modules projected these features into a shared latent space $R^{d}$, where the model was optimized using a contrastive learning objective.

### Image Embedding Module

Each input image feature $I_{t}\varepsilon R^{d_{i}}$ (for individual *t* where *d_i_* is the number of imaging features) was transformed into an embedding $e_{t,i}\varepsilon R^{d}$ through a series of transformations. First, the input *I_t_* is projected using two linear transformations: $h_{t,i}^{\left(1\right)}=I_{t}W_{1}+b_{1}$ and $h_{t,i}^{\left(2\right)}=W_{2}h_{1}+b_{2}$, where $W_{1}\varepsilon R^{d_{i}\times d}$ and $W_{2}\varepsilon R^{d\times d}$ were the weight matrices, and $b_{1}\varepsilon R^{d}$, $b_{1}\varepsilon R^{d}$ were the associated bias terms.

To introduce non‐linearity, the Gaussian Error Linear Unit (GELU)^[^ ^39^ ^]^ activation function was used: $h_{t,i}^{\left(3\right)}=\mathrm{GELU}\left(h_{t,i}^{\left(2\right)}\right)$. The activation output was then passed through an additional linear transformation, followed by dropout regularization: $h_{t,i}^{\left(4\right)}=Dropout\left(h_{t,i}^{\left(3\right)}W_{3}+b_{3}\right)$, where $W_{3}\varepsilon R^{d\times d}$ and $b_{3}\varepsilon R^{d}$ were the weight matrix and bias term, respectively. A residual connection combined this output with $h_{t,i}^{\left(1\right)}$: $z_{t,i}=h_{t,i}^{\left(4\right)}+h_{t,i}^{\left(1\right)}$. Finally, the resultant vector *z*_*t*,*i*_ was normalized to generate the image embedding: $e_{t,i}=\frac{z_{t,i}-\mu_{i}}{\sigma_{i}}$, where µ_*i*_ and σ_*i*_ were the mean and variance of *z*_*t*,*i*_.

### SNP‐Label Embedding Module

Similarly, SNP‐label input $s_{t}\varepsilon R^{d_{s}}$ was processed into an embedding $e_{s,i}\varepsilon R^{d}$ through a series of transformations. Specifically, the input *s_t_* was projected using two linear transformations: $h_{t,s}^{\left(1\right)}=s_{t}W_{1}^{\prime }+b_{1}^{\prime }$ and $h_{t,s}^{\left(2\right)}=h_{t,s}^{\left(1\right)}W_{2}^{\prime }+b_{2}^{\prime }$. Here, $W_{1}^{\prime }\varepsilon R^{d_{s}\times d}$ and $W_{2}^{\prime }\varepsilon R^{d\times d}$ were the weight matrices for the first and second linear layers, respectively, while $b_{1}^{\prime }\varepsilon R^{d}$ and $b_{2}^{\prime }\varepsilon R^{d}$ were the associated bias terms.

Then the GELU activation function was applied: $h_{t,s}^{\left(3\right)}=\mathrm{GELU}\left(h_{t,s}^{\left(2\right)}\right)$. After activation, another linear transformation and dropout were applied: $h_{t,s}^{\left(4\right)}=Dropout\left(h_{t,s}^{\left(3\right)}W_{3}^{\prime }+b_{3}^{\prime }\right)$, where $W_{3}^{\prime }\varepsilon R^{d\times d}$ were the weight matrices and $b_{3}^{\prime }\varepsilon R^{d}$ was the associated bias term. The residual connection combines $h_{t,s}^{\left(4\right)}$ was combined with the output of the first linear transformation $h_{t,s}^{\left(1\right)}$, i.e. $z_{t,s}=h_{t,s}^{\left(4\right)}+h_{t,s}^{\left(1\right)}$. The final SNP‐label embedding *e*_t,*s*_ was normalized: $e_{t,s}=\frac{z_{t,s}-\mu_{s}}{\sigma_{s}}$, where µ_*s*_ and σ_*s*_ were the mean and variance of *z*_*t*,*s*_.

### Loss Function

A contrastive loss aligns embeddings from the same individual while separating embeddings from different individuals. The similarity score between the image embedding and the SNP‐label embedding for the same individual was defined as: $sim\left(e_{t,i},\;e_{t,s}\right)=\frac{e_{t,i}\cdot e_{t,s}}{\tau }$, where τ is a temperature parameter that scales the similarity scores and · denotes the dot product between embeddings. *e*_t,*i*_ is the embedding derived from the image data of individual *t* and *e*_t,*s*_ is the embedding derived from the SNP‐label data of individual *t*. The contrastive loss is computed over a batch of *B* samples as: $L=\frac{1}{B}\sum_{t=1}^{B}-log\exp\left(sim(e_{t,i},\;e_{t,s})\right)/\sum_{t^{\prime }=1}^{B}\exp\left(sim(e_{t,i},\;e_{t^{\prime },s})\right)$.

### Inference Stage

In the inference stage, image embeddings were generated from the test set and passed into an ensemble voting classifier comprising Random Forest, SVM, and XGBoost. Each classifier makes a prediction *p_k_*, and the final output was computed via weighted voting: $\hat{y}=argmax_{c}\sum_{k=1}^{K}w_{k}*I\left(p_{k}=c\right)$, where *w_k_* denotes the classifier weights. $I(p_{i}=c)$ is an indicator function that equals 1 if the prediction *p_k_* corresponds to class *c*, and 0 otherwise. c ranges over all possible classes (AD, MCI, NC). The class *c* with the highest weighted sum was selected as the final prediction for each test image.

### Statistical Analysis—Pre‐Processing Data

This study utilized imaging and genetic datasets from two large‐scale cohorts: the Alzheimer's Disease Neuroimaging Initiative (ADNI) and the UK Biobank (UKB). Each subject was characterized by both neuroimaging features and single nucleotide polymorphism (SNP) profiles. SNPs associated with brain‐related traits and neurological disorders were curated from the GWAS Catalog.^[^ ^40^ ^]^ To ensure consistency, only SNPs present in both ADNI and UKB were retained. For UKB, pre‐computed image‐derived phenotypes (IDPs) were used, which included brain volumes, cortical thickness, and surface area measurements processed via standardized pipelines (Table S1, Supporting Information). For ADNI, raw T1‐weighted MRI scans were preprocessed using self‐supervised learning techniques inspired by iGWAS^[^ ^41^ ^]^ Preprocessing included spatial normalization, intensity normalization, and bias field correction, followed by latent imaging feature extraction.

### Statistical Analysis—Data Presentation

Model convergence was assessed through epoch‐wise learning curves displaying loss trajectories, accuracy evolution, and area under the receiver operating characteristic curve (AUC). Classification performance was evaluated using receiver operating characteristic (ROC) analysis with systematic comparison across unimodal (MRI‐only, SNP‐only) and multimodal (MRI+SNP) configurations. Genotype‐stratified imaging biomarker distributions were visualized using violin plots, revealing structural brain variations across genetic backgrounds and diagnostic categories. SHAP summary plots ranked features by global importance while encoding directionality and magnitude of effects. SHAP interaction analysis revealed relationships between genetic variants and neuroimaging features.

### Statistical Analysis—Sample Size

The ADNI dataset utilized in this analysis consisted of a total of 758 subjects, divided across three diagnostic categories to adequately capture clinical heterogeneity. Specifically, 214 subjects were diagnosed with AD, 180 subjects presented MCI, and 364 were classified as cognitively normal (CN) controls. In parallel, the UKB dataset incorporated a total of 261 subjects, comprising 61 AD patients and 200 CN controls. The UKB dataset served as an independent validation cohort, enhancing the generalizability and robustness of the findings.

### Statistical Analysis—Statistical Methods

Classification performance was quantified using metrics including AUC, accuracy, sensitivity, specificity, and cross‐entropy loss. Model interpretability was achieved using SHAP values, with interaction effects between SNPs and neuroimaging features quantified through SHAP interaction values. Pearson correlation analysis quantified relationships between model‐prioritized features and standardized clinical measures: Mini‐Mental State Examination (MMSE), Clinical Dementia Rating (CDR), and Functional Activities Questionnaire (FAQ). Statistical significance was determined using two‐tailed tests with α = 0.05. Correlation patterns were visualized using network diagrams.

### Statistical Analysis—Software used for Statistical Analysis

All analyses were performed using Python (version 3.8). Deep learning models were developed using PyTorch (v1.12+), while classification and performance evaluation were conducted with scikit‐learn (v1.1+), ensuring reproducibility and adherence to established machine learning standards. Model interpretability was facilitated by SHAP (v0.41+), providing insights into feature importance. Data processing and statistical analysis were carried out using NumPy (v1.21+) and Pandas (v1.4+), and visualizations were created with Matplotlib (v3.5+) and Seaborn (v0.11+) to support data presentation.

### Code Availability

Code can be found at https://www.github.com/QSong‐github/AlzCLIP.

## Conflict of Interest

The authors declare no conflict of interest.

## Author Contributions

Y.W. performed conceptualization, methodology, software development, and data curation. Q.W. conducted data analysis and contributed to methodology. M.Z. was responsible for data acquisition and statistical analysis. J.L. contributed to figure preparation and visualization. L.Y. assisted with result interpretation and project supervision. B.A. provided domain expertise and contributed to the interpretation. X.Z. oversaw project supervision, secured funding, and reviewed the manuscript. Q.S. led conceptualization, project administration, supervision, funding acquisition, and correspondence. All authors reviewed and edited the manuscript.

## Supporting information



Supporting Information

## Data Availability

Data used in the preparation of this article were obtained from the UK Biobank (https://www.biobank.ndph.ox.ac.uk/showcase/label.cgi?id=508) and Alzheimer's Disease Neuroimaging Initiative (ADNI) database (www.loni.ucla.edu/ADNI).
