# Supplementary material for: Integration of Genetic and Imaging Data for Alzheimer's Disease Diagnosis and Interpretation
Source: Adv Sci (Weinh). 2025 Aug 11;12(41):e07629. doi: 10.1002/advs.202507629 (PMC12591116; doi:10.1002/advs.202507629)
Supplement: Supplementary file 1 — Supporting Information [file ADVS-12-e07629-s001.docx]

**SUPPLEMENTARY INFORMATION**

**Integration of Genetic and Imaging Data for Alzheimer’s Disease Diagnosis and Interpretation**

Supplementary Figures ---------------- 3-4

Supplementary Table ---------------- 5-7

Supplementary Notes ---------------- 8

Table of Contents

[Supplementary Fig. 1 3](#_Toc202971021)

[Supplementary Fig. 2 4](#_Toc202971022)

[Supplementary Table 1 5](#_Toc202971023)

[Supplementary Table 2 7](#_Toc202971024)

[Note 1: 8](#_Toc202971025)

**Supplementary Figure 1. Training performance curves for the ADNI dataset (n=758).** Training dynamics across 600 epochs for three experimental configurations: MRI-only (left column), SNP-only (center column), and MRI+SNP (right column). Upper row shows AUC progression over training epochs for AlzCLIP (red line) compared to baseline methods including 3D-DAM, 3D-ResNet, CNN (for MRI), SVM, Random Forest, XGBoost (for SNP), and Gene-SGAN, ViT-XGBoost, RACF (for combined modalities). Middle row displays accuracy curves over training epochs with identical model comparisons and color coding. Lower row presents training loss curves demonstrating convergence behavior across all methods. AlzCLIP consistently demonstrates superior and stable performance across all metrics and modalities. Data represent training set performance without error bars or statistical testing indicated. Final epoch performance values can be read from curve endpoints, with AlzCLIP achieving highest AUC and accuracy.

**Supplementary Figure 2**: **Training performance curves for the UKB dataset (n=261).** Training dynamics across 600 epochs for three experimental configurations: MRI-only (left column), SNP-only (center column), and MRI+SNP (right column). Upper row shows AUC progression over training epochs for AlzCLIP (red line) compared to baseline methods including 3D-DAM, 3D-ResNet, CNN (for MRI), SVM, Random Forest, XGBoost (for SNP), and Gene-SGAN, ViT-XGBoost, RACF (for combined modalities). Middle row displays accuracy curves over training epochs with identical model comparisons and color coding. Lower row presents training loss curves demonstrating convergence behavior across all methods. AlzCLIP consistently demonstrates superior and stable performance across all metrics and modalities. Data represent training set performance without error bars or statistical testing indicated. Final epoch performance values can be read from curve endpoints, with AlzCLIP achieving highest AUC and accuracy.

**Supplementary Table 1**

**Supplementary Table 2**

**SUPPLEMENTARY NOTES**

**Note 1: AlzCLIP demonstrates superior performance in AD diagnosis**

As shown in **Supplementary Figure 1**, AlzCLIP achieved superior performance across all experimental settings in the ADNI cohort. In the MRI-only configuration, AlzCLIP obtained a final accuracy of 0.62, sensitivity of 0.68 and specificity of 0.56, outperforming comparative imaging-based models, including 3D-DAM (accuracy: 0.48, sensitivity: 0.65, specificity: 0.31), 3D-ResNet (accuracy: 0.47, sensitivity: 0.62, specificity: 0.32) and CNN (accuracy: 0.46, sensitivity: 0.70, specificity: 0.36). In the SNP-only setting, AlzCLIP achieved a final accuracy of 0.60, sensitivity of 0.72, and specificity of 0.48, surpassing other models such as CNN (accuracy: 0.46, sensitivity: 0.70, specificity: 0.36), SVM (accuracy: 0.58, sensitivity: 0.65, specificity: 0.51), RF (accuracy: 0.57, sensitivity: 0.69, specificity: 0.45), and XGBoost (accuracy: 0.56, sensitivity: 0.63, specificity: 0.49). The highest predictive capability was observed under the combined MRI+SNP scenario, where AlzCLIP achieved a final accuracy of 0.81, sensitivity of 0.80, and specificity of 0.76, substantially exceeding the multimodal baseline Gene-SGAN (accuracy: 0.78, sensitivity: 0.80, specificity: 0.76), ViT-XGBoost (accuracy: 0.70, sensitivity: 0.75, specificity: 0.65), RACF (accuracy: 0.65, sensitivity: 0.78, specificity: 0.52), as well as SVM (accuracy: 0.65, sensitivity: 0.67, specificity: 0.63), Random Forest (accuracy: 0.64, sensitivity: 0.71, specificity: 0.57), and XGBoost (accuracy: 0.63, sensitivity: 0.68, specificity: 0.58).

This performance superiority was consistently confirmed in the UKB cohort, as demonstrated in **Supplementary Figure 2**. Under the MRI-only condition, AlzCLIP achieved a final accuracy of 0.73, sensitivity of 0.76, and specificity of 0.70, clearly surpassing imaging-based methods such as 3D-DAM (accuracy: 0.67, sensitivity: 0.71, specificity: 0.63), 3D-ResNet (accuracy: 0.67, sensitivity: 0.68, specificity: 0.64) and CNN (accuracy: 0.70, sensitivity: 0.68, specificity: 0.72). Similarly, in the SNP-only scenario, AlzCLIP maintained robust performance with a final accuracy of 0.65, sensitivity of 0.78, and specificity of 0.52, outperforming conventional SNP-based approaches including CNN (accuracy: 0.62, sensitivity: 0.70, specificity: 0.43), SVM (accuracy: 0.63, sensitivity: 0.74, specificity: 0.52), RF (accuracy: 0.52, sensitivity: 0.70, specificity: 0.34), and XGBoost (accuracy: 0.57, sensitivity: 0.65, specificity: 0.49). The combined MRI+SNP configuration again delivered the strongest predictive outcomes, where AlzCLIP reached a final accuracy of 0.79, sensitivity of 0.82, and specificity of 0.76, outperforming the multimodal baseline Gene-SGAN (accuracy: 0.73, sensitivity: 0.74, specificity: 0.72), ViT-XGBoost (accuracy: 0.78, sensitivity: 0.79, specificity: 0.77), RACF (accuracy: 0.75, sensitivity: 0.76, specificity: 0.74), as well as SVM (accuracy: 0.67, sensitivity: 0.68, specificity: 0.66), Random Forest (accuracy: 0.64, sensitivity: 0.72, specificity: 0.56), and XGBoost (accuracy: 0.68, sensitivity: 0.65, specificity: 0.62).
